# Impact of Type 2 Diabetes Mellitus on the Incidence and Outcomes of COVID-19 Needing Hospital Admission According to Sex: Retrospective Cohort Study Using Hospital Discharge Data in Spain, Year 2020

**DOI:** 10.3390/jcm11092654

**Published:** 2022-05-09

**Authors:** Jose M. de Miguel-Yanes, Rodrigo Jimenez-Garcia, Javier de Miguel-Diez, Valentin Hernández-Barrera, David Carabantes-Alarcon, Jose J. Zamorano-Leon, Ricardo Omaña-Palanco, Ana Lopez-de-Andres

**Affiliations:** 1 Internal Medicine Department, Hospital General Gregorio Marañón, Universidad Complutense de Madrid, Instituto de Investigación Sanitaria Gregorio Marañón (IiSGM), 28007 Madrid, Spain; josemaria.demiguel@salud.madrid.org; 2 Department of Public Health & Maternal and Child Health, Faculty of Medicine, Universidad Complutense de Madrid, Instituto de Investigación Sanitaria Gregorio Marañón (IdISSC), 28040 Madrid, Spain; dcaraban@ucm.es (D.C.-A.); josejzam@ucm.es (J.J.Z.-L.); romana@ucm.es (R.O.-P.); anailo04@ucm.es (A.L.-d.-A.); 3 Respiratory Department, Hospital General Gregorio Marañón, Universidad Complutense de Madrid, Instituto de Investigación Sanitaria Gregorio Marañón (IiSGM), 28007 Madrid, Spain; javier.miguel@salud.madrid.org; 4 Preventive Medicine and Public Health Teaching and Research Unit, Health Sciences Faculty, Universidad Rey Juan Carlos, 28922 Alcorcon, Spain; valentin.hernandez@urjc.es

**Keywords:** COVID-19, type 2 diabetes mellitus, hospitalization, incidence, mortality

## Abstract

(1) Background: To analyze incidence and in-hospital mortality (IHM) of COVID-19 needing hospital admission in Spain (2020) in patients with T2DM. (2) Methods: We conducted a retrospective cohort study. Using the Spanish Register of Specialized Care-Basic Minimum Database we estimated age-adjusted incidence rates (IR). (3) Results: We included 203,488 patients (56.77% men), of whom 45,620 (22.41%) had T2DM. Age-adjusted IRs/1000 for men with and without T2DM was 12.90 and 5.87, respectively (IRR 2.20; 95% CI 2.18–2.22; *p* < 0.001), and for women with and without T2DM was 9.23 and 4.27, respectively (IRR 2.16; 95% CI 2.13–2.19; *p* < 0.001). Crude IHM was 23.86% in people with T2DM, and 15.94% in non-T2DM people (*p* < 0.001). After matching, intensive-care admission (7.37% vs. 6.15%; *p* < 0.001) and IHM (23.37% vs. 20.41%; *p* < 0.001) remained higher in women with T2DM. After matching, IHM among T2DM men was 1.5% higher than among non-T2DM men (24.27% vs. 22.72%; *p* < 0.001). Men with T2DM had a 34% higher IHM than women with T2DM. Prevalent T2DM increased IHM among women (1.09; 95% 1.03–1.16) and men (1.05; 95% 1.01–1.10). (4) Conclusions: Incidence rates of COVID-19 needing hospital admission were higher in men vs. women, and for people with T2DM vs. non-T2DM. Men had higher IHM beside T2DM status. Prevalent T2DM was associated with higher IHM for both sexes.

## 1. Introduction

By 13 February 2022, more than 410 × 10^6^ confirmed cases of COVID-19 cases have been reported, with a death toll of more than 5.8 × 10^6^ fatalities [1]. Early studies published after the first wave of the pandemic showed a high prevalence of type 2 diabetes mellitus (T2DM) among patients who needed hospital admission and underscored the prognostic impact of diabetes and other comorbidities on in-hospital mortality (IHM) [2]. It was proposed that hyperglycemia might modulate immune and inflammatory responses, thus predisposing patients to severe COVID-19 and possible lethal outcomes [3]. On the opposite, use of steroids has been reported to improve survival under specific conditions during hospital admission for COVID-19 despite the marked hyperglycemia that the use of steroids at high doses often entails [4].

Along the pandemic, larger studies have been released. A meta-analysis by Corona et al., which included more than 35,000 patients, showed an association between diabetes and IHM [5]. A nationwide analysis in England showed that T2DM was independently associated with significantly increased odds of in-hospital death with COVID-19 [6]. However, lack of adjustment for a full set of variables frequently poses limitations in observational studies.

Using records of the hospital admissions in Spain for the year 2020, in this study we aimed to: (1) assess the effect of T2DM on the incidence of hospital admissions with COVID-19 according to sex; (2) analyze the effect of T2DM on the IHM among patients hospitalized with COVID-19 according to sex; (3) find which sociodemographic and clinical conditions were associated with IHM among T2DM men and women hospitalized with COVID-19.

## 2. Materials and Methods

### 2.1. Study Design

We have conducted a retrospective cohort study. Data were obtained from the Spanish National Hospital Discharge Database (SNHDD). The SNHDD is an administrative database managed by the Spanish Ministry of Health (SMH) that collects information from all hospitals, public and private, in Spain. According to the Spanish legislation, all Spanish hospitals must fulfill and send data to the SMH with annual periodicity [7,8].

The information collected by the SNHDD is age, sex, place of residence, dates of admission and discharge, discharge destination (home, other social or medical institution, voluntary discharge, or decease), primary diagnosis, secondary diagnoses (up to 19), and therapeutic and diagnostic procedures conducted during the hospitalization (up to 20). Coding in the SNHDD is executed with the International Classification of Disease 10th version (ICD10). Details on the SNHDD can be found elsewhere [7,8].

As the first cases of confirmed COVID-19 were detected in Spain on March 2020 the study period runs from 1 March to 31 December 2020.

### 2.2. Study Population and Participants

Our study population included persons aged 30 year or over admitted to a hospital with a diagnosis of COVID-19 over the last ten months of 2020. The definition of used is the recommended by the SMH to codify this disease using ICD10 (See Table S1) [9,10,11] during 2020.

We defined as exposed cohort those patients who suffered T2DM when admitted to the hospital. A participant was classified as a person with T2DM if a code for T2DM (ICD10 E11) is recorded in any diagnosis position (1–20) along with a “Present On Admission” (POA) indicator of “Yes”. The POA allows us to figure out which conditions existed before the patient came to the hospital (Table S1).

The unexposed cohort included all admissions without a code for T2DM.

Exclusion criteria for both cohorts were (i) persons with missing data for age, sex, place of residence, dates of admission or discharge, and discharge destination; (ii) persons with a code for T1DM (ICD10 E10) in any diagnosis field, and (iii) if the same individual was admitted more than once during the study period with COVID-19, only the first episode was analyzed.

The study population was stratified by sex for description and analysis.

### 2.3. Matching

For each person with T2DM (exposed cohort) we selected a non-T2DM subject (un-exposed cohort) with identical sex, age, region of residence, and month of admission.

The reason to match using the region of residence is that previous studies conducted in Spain have shown that the evolution of the COVID-19 pandemic has been quite heterogeneous across the Spanish provinces, showing relevant differences in both incidence and severity [12,13,14].

The need to match by month is justified because it has been described that the case-fatality from COVID-19 during the second epidemic wave in Spain (June–December 2020) improved compared to the first wave (March–May 2020) [15,16,17].

Possible explanations for the differences in the incidence between the Spanish regions include, among others, population density, age, and mobility, as well as varying policies and control measures implemented by authorities at the local level [12,13,14,18].

The lower mortality in the second wave may be due to several reasons: (1) Overall improvements in medical skills in the last months of 2020, including better treatment strategies; (2) better health care organization and, as a result, avoidance of a system overload; (3) a change in patient characteristics; (4) the presence of viral variants with less pathogenicity [15,16,17].

If more than one unexposed person was available for an exposed case, the person with the closest date of admission was included.

### 2.4. Variables

The main outcome variables of this investigation were the incidence of hospital admission and the IHM according to the presence of T2DM and sex.

To estimate the incidence of hospitalizations in the study cohorts we used as a denominator the weighted number of persons with and without T2DM in Spain, by age groups and sex, according to the self-reported prevalence of physician diagnosed diabetes among participants of the European Health Interview Survey for Spain (EHISS2020) conducted from July 2019 to July 2020 [19].

The IHM is defined as the proportions of hospitalized patients that died during their hospital admission.

Secondary outcome variables were admission (yes/no) to the intensive care unit (ICU), duration of stay at the ICU, and total length of hospital stay (LOHS).

Study covariates used for matching were region of residence (Spain is divided into 19 regions) and the month of admission.

To assess the global comorbidity, the mean number of conditions included in the Charlson comorbidity index (CCI) was calculated using the algorisms previously proposed by Sundararajan et al. and Quan et al. [20,21].

Specific conditions analyzed were obesity, myocardial infarction, congestive heart failure, peripheral vascular disease, cerebrovascular disease, dementia, chronic obstructive pulmonary disease (COPD), rheumatoid disease, mild/moderate/severe liver disease, chronic renal disease, and cancer or metastatic cancer. Procedures included the use of non-invasive and invasive mechanical ventilation. The clinical conditions and therapeutic procedures analyzed and the ICD10 codes used to name them are described in Table S1.

### 2.5. Statistical Methods

Incidence rates of COVID-19 admissions were calculated for both cohorts according to sex and age groups. The direct method was used to obtain age-adjusted incidence rates using the total Spanish population as a standard. Incidence rate ratios (IRR) with 95% confidence intervals (95% CI) were estimated.

The descriptive analysis was performed with the calculation of means with standard deviation or medians with interquartile range for quantitative variables, and with absolute and relative frequencies, expressed as percentages, for qualitative variables.

The statistical methods applied for the comparison of means, medians, and proportions, between the study subpopulations, were the Student’s T test, the Wilcoxon–Mann–Whitney test, and the chi square test, respectively.

Multivariable logistic regression models were constructed to evaluate which of the study variables were independently associated with IHM in each of the study subpopulations. This statistical method was applied following the recommendations proposed by Hosmer et al. [22]. Two-way interactions were examined.

### 2.6. Sensitivity Analyses

Even if we matched for relevant variables that are associated with the IHM, the effect of other confounding variables could not be controlled. Therefore, to assess the effect of T2DM in the IHM among women, men and persons of both sexes hospitalized with COVID-19, we constructed three multivariable logistic regression models using the matched sub-populations.

Stata 14 was the software used for matching and data analysis. A significance level of *p* < 0.05 (two tails) was considered statistically significant.

### 2.7. Ethical Aspects

The SNHDD database can be requested from the SMH at the following link [23]. The authorities of the Ministry conduct an evaluation of the proposal and if they consider it adequate from the scientific and ethical point of view, they send the anonymized records. Therefore, the study protocol has not been evaluated by an ethics committee and, as this is an administrative database, informed consent was not needed from the participants.

## 3. Results

In Spain, according to the SNHDD, in the last ten months of the year 2020, the total number of hospital admission with COVID-19 was 218,736. After inclusion and exclusion criteria were applied the study population included 203,488 patients. Of them, 115,512 (56.77%) were men (Table 1). The total prevalence of T2DM was 22.41% (*n* = 45,620), with a significantly higher proportion among men than women (23.69% vs. 20.74%; *p* < 0.001).

**Figure 1:**
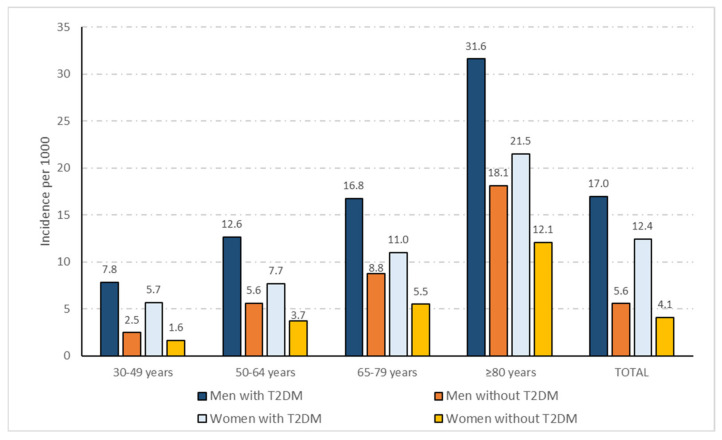
Incidence rates of hospital admission with COVID-19 per 1000 inhabitants with and without T2DM according to sex and age groups.

In Figure 1 are shown the incidence rates for hospital admission with COVID-19 among men and women with and without T2DM according to sex and age groups. The total crude incidence rates were 17.0 per 1000 men with T2MD and 12.4 per 1000 women with T2DM (*p* < 0.001). Incidence rates were higher among men and women with T2DM than among non-T2DM men and women for all age groups. Beside the presence of T2DM, men had higher incidence rates than women in all age groups. Using the direct method, we obtained age-adjusted incidence rates for men with and without T2DM of 12.90 and 5.87, respectively (IRR 2.20; 95% CI 2.18–2.22; *p* < 0.001). Among women the age-adjusted rates were significantly lower than for men (9.23 per 1000 women with T2DM and 4.27 per 1000 women without T2DM; *p* < 0.001) and the IRR showed a similar value (2.16; 95% CI 2.13–2.19; *p* < 0.001) than the one found for men. No significant difference in the IRR was found between sexes, which means that the increase in the risk of hospitalization was the same for men and women with T2DM when compared with men and women without T2DM.

The distribution by sociodemographic characteristics, clinical variables, and in-hospital outcomes of patients hospitalized with COVID-19 in Spain in the year 2020 according to diabetes status can be seen in Table 1. The proportion of women was significantly higher among those without T2DM (44.17% vs. 40%; *p* < 0.001). Patients with T2DM were significantly older than those without T2DM (73.35 years vs. 65.99 years; *p* < 0.001) and the mean CCI was also higher with figures of 0.88 and 0.52, respectively (*p* < 0.001). All of the chronic conditions analyzed were more often recorded among people with T2MD than non-T2DM patients.

The use of non-invasive and invasive mechanical ventilation was codified more frequently among patients with T2DM. The proportions of hospitalized COVID-19 subjects admitted to the ICU were 10.45% for those with T2DM and 9.67% for those without T2DM (*p* < 0.001) and the median of days at the ICU was longer for non-T2DM patients (11 days vs. 10 days; *p* < 0.001). On the other hand, the total LOHS was higher for those with T2DM (10 days vs. 9 days; *p* < 0.001). The IHM among the population with T2DM was 23.86%, decreasing significantly to 15.94% for those without this condition (*p* < 0.001).

In Table 2 can be seen the distribution of study covariates and hospital outcomes, before and after matching, for women hospitalized for COVID-19 in Spain in the year 2020 according to T2DM status. We could match 95.65% of women with T2DM with a non-T2DM woman (17,457/18,250). Before matching, women with T2DM were older (75.68 years vs. 67.84 years; *p* < 0.001), and had higher mean CCI and prevalence of all of the clinical conditions described except for COPD, rheumatoid disease and cancer or metastatic cancer. The use of mechanical ventilation, admission to the ICU, LOHS, and IHM (23.17% vs. 14.83%; *p* < 0.001) showed higher values among women with T2DM when compared to those without T2DM.

After matching the differences in the mean CCI, most chronic conditions and use of mechanical ventilation were reduced but were still significantly higher among women with T2DM. Additionally, the proportion of women admitted to the ICU (7.37% vs. 6.15%; *p* < 0.001), the LOHS (9 days vs. 8 days; *p* < 0.001), and the IHM (23.37% vs. 20.41%; *p* < 0.001) remained higher for women with T2DM.

In Table 3 is shown the distribution of the study variables before and after matching for men with and without T2DM. The proportion of men with T2DM to matched men reached 95.98%. As found among women, before matching, men with T2DM had a higher mean age and CCI than non-T2DM men (71.80 years vs. 64.52 years and 0.92 vs. 0.54; *p* < 0.001 for both). The prevalence of every chronic condition was significantly lower among men without T2DM. Additionally, fewer patients without T2DM received non-invasive mechanical ventilation (5.6% vs. 6.82%; *p* < 0.001). The median LOHS (9 days vs. 8 days; *p* < 0.001) and the IHM (24.32% vs. 16.82%; *p* < 0.001) were higher among those men with T2DM.

After matching, the differences between men with and without T2DM narrowed and became not significant for dementia, COPD, rheumatoid disease, cancer, metastatic cancer, non-invasive mechanical ventilation, and LOHS. However, after matching, the IHM among men with T2DM was still 1.5% higher than among men without T2DM (24.27% vs. 22.72%; *p* < 0.001).

The description of the IHM according to study variables among men and women with and without T2DM is shown in Table 4. Men died in the hospital in a higher proportion than women both when they suffered T2DM and not. IHM rose with age in all study subpopulations. Additionally, in all patients, beside sex and the presence of T2DM, the use of any mechanical ventilation (IHM > 40%) and being admitted to the ICU (>36%) were associated with higher IHM. Among women with T2DM the conditions associated with the highest mortality were cancer or metastatic cancer (40.15%), dementia (39.53%), congestive heart failure (38.79%), and renal disease (35.72%). These were the same most frequent chronic diseases among women without T2DM. Men with dementia and T2DM had an IHM of 45.39%; equivalent figures for congestive heart failure, cancer, or metastatic cancer and renal disease were 40.77%, 37.05, and 37.04%, respectively. As seen among women, the differences in the mortality rates among men without T2DM and among men with T2DM did not reach statistical significance for most comorbid conditions.

The results of multivariable analysis performed to identify those variables associated with IHM in patients hospitalized with COVID-19 in Spain, in 2020, according to sex and T2DM status can be seen in Table 5. For all of the study subgroups the risk of dying in the hospital increased as age rose and was significantly higher among those who had a code recorded for congestive heart failure, cerebrovascular disease, dementia, renal disease, cancer, metastatic cancer, non-invasive mechanical ventilation, invasive mechanical ventilation, and admission to the ICU. For men with T2DM, suffering concomitant myocardial infarction or peripheral vascular disease were risk factors for IHM.

When the databases with men and women with T2DM were joined we saw that men had a 34% higher mortality risk than women (OR 1.34, 95% CI 1.27–1.42).

Finally, in Table 6 are shown the results of the sensitivity analysis. The multivariable logistic regression confirmed that after controlling for covariables the presence of T2DM prior to hospital admission increased the risk of dying during the hospitalization among women (1.09; 95% 1.03–1.16), men (1.05; 95% 1.01–1.10), and both sexes (1.06 95%; 1.03–1.10).

## 4. Discussion

Here we found higher incidence rates of COVID-19 patients needing hospital admission among men than among women. Indeed, higher incidence rates, higher rates of ICU admission, and a higher IHM have been observed for men vs. women globally [24]. Differences in the immune responses of each sex, the role of sex hormones, or gender-related risk factors of progression of the disease have been suggested to explain these differences [25].

We could also see that the age-adjusted IRR was slightly over 2 for men and women with T2DM, showing that in both sexes the incidence of hospital admission for COVID-19 was twice higher than for subjects without T2DM. This increased hospitalization risk in people with T2DM has been previously reported [26]. Several possible mechanisms have been highlighted to explain these findings. Hyperglycemia might support viral proliferation [27]. Furthermore, virally induced inflammation increases insulin resistance, which hampers the optimal metabolic control of patients with T2DM, especially when steroids are used at high doses [28]. Moreover, individuals with impaired glucose tolerance or diabetes mellitus have reduced natural killer cell activity [29], which could made them more susceptible to more severe COVID-19. In our study, patients with T2DM were older and had more comorbid conditions, but the adjustment for covariates done in our study should have minimized the confounding effect of these predefined clinical factors.

We observed that the IHM among the population with T2DM was one third higher (23.86% versus 15.94%) than among the non-T2DM population. Additional factors of increased risk of complications and damage to vital organs in patients with T2DM could be glucotoxicity, endothelial injury by inflammation, oxidative stress, and cytokine production [30]. Moreover, an increased venous thromboembolic risk and the potentially deleterious effects exerted by many of the treatments tested during the first waves of SARS-CoV-2 might have contributed to more severe forms of COVID-19 in the population with T2DM [3,31].

After matching, the proportion of women admitted to the ICU, women undergoing mechanical ventilation, and the LOHS remained higher for women with T2DM. Additionally, IHM was higher among women with T2DM than among women without T2DM. Among men, the IHM in the group with T2DM was still 1.5% higher than among non-T2DM men (24.27% vs. 22.72%), although differences in the proportion of ICU admission, mechanical ventilation, and the LOHS were no longer significant. All of these results were confirmed by sensitivity analyses. Attending to the data shown for women, we might hypothesize that a higher severity in T2DM women could help explain the increased IHM in this subpopulation. Yet, men with T2DM had a higher IHM than men without T2DM and this result was apparently not explained by markers of a higher clinical severity, since the differences in this regard between men with and without T2DM were non-significant.

Multivariable analyses to identify factors associated with IHM showed that in all of the study subgroups, with and without T2DM, the risk of dying in the hospital increased as age rose and was significantly higher among those who had a code recorded for congestive heart failure, cerebrovascular disease, dementia, renal disease, cancer, or metastatic cancer, and for procedures such as non-invasive mechanical ventilation, invasive mechanical ventilation, and admission to the ICU. For men with T2DM, prior myocardial infarction or peripheral vascular disease were risk factors for IHM, too. Earlier in the pandemic, the impact of comorbidities had been reported by different researchers, as in the meta-analysis by Singh et al. [32]. Even later, some authors have continued to claim that advanced age and comorbidities rather than diabetes itself were associated with increased in-hospital mortality in COVID-19 patients [33]. Comorbidities are considered important risk factors of progression to more severe forms of COVID-19. In fact, their presence has been used to identify target populations for their inclusion in randomized clinical trials of efficacy of newer treatments for the infection or to test expanded indications of certain formerly evaluated therapies [34,35].

When the databases with men and women with T2DM were joined, we found that men had a 34% higher in-hospital mortality risk than women. Higher IHM in men has been reported previously even after additional clinical factors were accounted for [36]. Beyond the recurrent arguments of the role of sex hormones and unmeasured risk factors [37,38], the idea of a male sex-differential excess all-cause mortality in situations of overall excess mortality, albeit provocative, has been defended by some investigators [39]. This argument suggests that there is a consistent pattern of higher excess mortality among males in periods with overall excess mortality, reproducible in other periods of high mortality, such as some of the past influenza seasons.

In our work, the presence of T2DM prior to hospital admission increased the risk of dying during the hospitalization among women, among men and when both sexes were combined. As stated in the background section, some early research could not find an association between prevalent T2DM and IHM in people admitted with COVID-19. We are now supporting this idea of a higher IHM associated with T2DM with the current study.

As commented before, most reports suggest the poorer outcome of people with diabetes who suffer COVID-19 [5,6,26,32]. In fact, very recently, Kastora et al. conducted a meta-analysis to assess the effect of diabetes on COVID-19 outcomes. A total of 158 observational studies were analyzed including a total of 270,212 participants, which originated from studies conducted in the European Union (22), far east (90), middle east (16), and from America (30). Pooled results showed that patients with diabetes were at a higher risk of COVID-19-related mortality (OR 1.87; 95% CI 1.61–2.17), ICU admissions (OR 1.59; 95% CI 1.15–2.18), and mechanical ventilation requirements (OR 1.44; 95% CI 1.20, 1.73) [40].

In Europe and other non-European countries, several studies have used national data or a methodology in line with our study to assess the effect of diabetes on the incidence and outcomes of COVID-19 [41,42,43,44,45,46].

In Austria, Aziz et al. analyzed data from 40,602 COVID-19 patients hospitalized from March 2020 to March 2021, provided by the Austrian National Public Health Institute, to assess the impact of diabetes on ICU admission and IHM [41]. The prevalence of diabetes was 13.2% (*n* = 4971), much lower than our results (22.41%). The crude IHM was significantly higher among those with than without diabetes (18.8% vs. 15.8%; *p* < 0.001) and this difference was also significant after propensity score matching (PSM) (18.8% vs. 17.8%; *p* = 0.028) but became non-significant after multivariable logistic regression (OR: 1.08, 95% CI: 0.97–1.19, *p* = 0.146). Compared with Austrian patients, the IHM found in our country is higher for those with diabetes and similar for those without this condition (23.86% vs. 15.94; *p* < 0.001) and in Spain this difference remained significant after multivariable regression (OR 1.06; 95% CI 1.03–1.10). The distribution by age, sex, and the prevalence of comorbid conditions could justify these results. However, we agree with the Austrian data reporting that diabetes was associated with a higher odds of ICU admissions and that old age, male sex, and comorbidities were significantly associated with IHM after multivariable adjustment [41].

In England, a whole-population study was conducted assessing risks of in-hospital death with COVID-19 between 1 March and 11 May 2020. After adjusting by age, sex, deprivation, ethnicity, geographical region, and previous hospital admissions with coronary heart disease, cerebrovascular disease, or heart failure, the ORs for IHM were 2.86 (95% CI 2.58–3.18) for type 1 diabetes and 1.80 (95% CI 1.75–1.86) for T2DM [6]. The much higher OR obtained in England compared to our results could be explained by the different methods used and as commented by the authors, because a high number of comorbid conditions were not included in the English investigation (BMI, hypertension, and kidney disease among other potential confounders) [6].

In the United States, a retrospective cohort study utilized administrative claims data from the UnitedHealth Group Clinical Discovery Database, a nationwide database, to evaluate the risk of COVID-19 hospitalization and mortality among people with T2DM [42]. As reported by us, the risks of hospital admission and IHM with COVID-19 among people with T2DM were higher than those for those without T2DM. The proportion of patients with T2DM that died in the hospital was 23.31%, a figure very similar to ours (23.86%). We also agree with the finding that age, male sex, and cardiovascular conditions were associated with IHM among people with T2DM hospitalized with COVID-19 [42]. In the same country, analyses were conducted on patients with an ICD-10 diagnosis of T2DM and COVID-19 admitted to any Northwell Health System hospital in the New York area between 1 January and 31 May 2020 [43]. Among the 4413 patients analyzed, the overall IHM was 24.78% and, as seen in previous studies and in our investigation, male gender, older age, and ventilation use were associated with increased mortality after multivariable logistic regression [41,42,43]. The role of ventilation as a risk factor for mortality was also found among men and women with T2DM in our study [43].

In Turkey, using national data from the Ministry of Health National Electronic Database, patients with T2DM (*n* = 9213) were matched by age and sex using PSM with a group without diabetes (*n* = 9213). Compared with the group without T2DM, the proportion of prolonged hospital stays, ICU admission, intubation, and death were significantly higher [44]. These results are very similar to ours even if the overall IHM was much lower, at 13.6% vs. 8.7%, compared to 23.86% vs. 15.94% in our investigation. However, the median age among the Turkish T2DM patients was 61 years compared to a mean of 73 years found in our investigation, a difference that could explain the gap in the IHM [44].

In Korea, two studies using National databases reported significantly worse hospital outcomes in patients with T2DM [45,46]. Moon et al., after adjusting for many comorbid conditions, obtained an adjusted OR for IHM of 2.659 (95% CI 1.896–3.729) for people with diabetes compared to those without this condition. In this investigation, like in ours, when the population was stratified by sex the association remined significant for men and women [45]. Additionally, in Korea, You et al. found that COVID-19 positive patients with T2DM had poorer clinical outcomes with higher risk of ICU admission (OR 1.59; 95% CI 1.02–2.49) and in-hospital mortality (OR 1.90; 95% CI 1.13–3.21) than those without diabetes [45]. In these studies conducted in Korea, male sex and older age predicted worse outcomes among T2DM patients [45,46].

The large sample size—with data from over 203,000 episodes of COVID-19 hospital admissions—and the widespread coverage of the Spanish population by the SNHDD (>95% of all hospital admissions) gives strength to the results reported here. 

### Limitations

We should point out the following limitations: We considered, for incidence calculations, that all persons with self-reported prevalence of physician diagnosed diabetes interviewed in the EHSS2020 had T2DM. This may slightly overestimate the prevalence of this condition and therefore the actual incidence of T2DM hospitalization would be underestimated. According to recent data, in Spain, over 96% of all diabetes is type 2 [47]. We could not differentiate between people admitted for COVID-19 and people who had been hospitalized for other reasons and developed COVID-19 during their hospital stay. Our data source is an administrative database supported by the information that physicians keep in the discharge report, which depends on manual coding on behalf of the administrative staff. Despite a pair-matching process that most likely contributed to attenuate sex-related differences in baseline characteristics and clinical variables, a complete elimination of residual confounding is difficult to achieve in observational case-control studies.

## 5. Conclusions

In our study, we saw that incidence rates of Spanish COVID-19 patients needing hospital admission were higher in men than in women, as well as in people with T2DM vs. people without T2DM. IHM increased with age and prevalent comorbidities. Men had a significantly higher IHM than women. Prevalent T2DM was associated with increased IHM among women, men, and in both sexes combined. Additional research is needed to fully understand the complex interaction between sex, T2DM and COVID-19.

## Figures and Tables

**Table 1:** Sociodemographic and clinical characteristics and in-hospital outcomes of patients hospitalized with COVID-19 in Spain, 2020, according to diabetes status.

	T2DM	No T2DM	*p*-Value
N (%)	45,620 (22.41)	157,868 (77.59)	<0.001
Sex women n (%)	18,250 (40)	69,726 (44.17)	<0.001
Age, mean (SD)	73.35 (12.58)	65.99 (16.36)	<0.001
30–49 years, n (%)	1948 (4.27)	28,901 (18.31)	<0.001
50–64 years, n (%)	9200 (20.17)	45,692 (28.94)	<0.001
65–79 years, n (%)	18,104 (39.68)	43,744 (27.71)	<0.001
≥80 years, n (%)	16,368 (35.88)	39,531 (25.04)	<0.001
CCI index, mean (SD)	0.88 (1)	0.52 (0.81)	<0.001
Obesity, n (%)	8065 (17.68)	15,147 (9.59)	<0.001
Myocardial infarction, n (%)	2767 (6.07)	4056 (2.57)	<0.001
Congestive heart failure, n (%)	5779 (12.67)	10,030 (6.35)	<0.001
Peripheral vascular disease, n (%)	2355 (5.16)	3729 (2.36)	<0.001
Cerebrovascular disease, n (%)	2639 (5.78)	4749 (3.01)	<0.001
Dementia, n (%)	3367 (7.38)	8288 (5.25)	<0.001
COPD, n (%)	6977 (15.29)	20,681 (13.1)	<0.001
Rheumatoid disease, n (%)	1002 (2.2)	3128 (1.98)	0.004
Mild Moderate/severe liver disease, n (%)	2985 (6.54)	6404 (4.06)	<0.001
Renal disease, n (%)	9242 (20.26)	12,832 (8.13)	<0.001
Cancer, or Metastatic cancer, n (%)	2445 (5.36)	7136 (4.52)	<0.001
Non-invasive mechanical ventilation, n (%)	2799 (6.14)	7406 (4.69)	<0.001
Invasive mechanical ventilation, n (%)	3391 (7.43)	10,925 (6.92)	<0.001
Admission to ICU, median (IQR)	4769 (10.45)	15,258 (9.67)	<0.001
Day in ICU median (IQR)	10 (15)	11 (16)	<0.001
LOHS, median (IQR)	9 (10)	8 (8)	<0.001
IHM, n (%)	10,886 (23.86)	25,165 (15.94)	<0.001

T2DM: type 2 diabetes mellitus; CCI: Charlson comorbidity index; COPD: chronic obstructive pulmonary disease; ICU Intensive care unit; IQR: Inter quartile range; LOHS: length of hospital stays; IHM: in-hospital mortality.

**Table 2:** Distribution of study covariates and hospital outcomes, before and after matching, for women hospitalized for COVID-19 in Spain in the year 2020 according to T2DM status.

	Before Matching			After Matching *		
	T2DM	No T2DM	*p*-Value	T2DM	No T2DM	*p*-Value
N (%)	18,250 (20.74)	69,726 (79.26)	<0.001	17,457 (50)	17,457 (50)	Matched 1:1
Age, mean (SD)	75.68 (12.67)	67.84 (16.87)	<0.001	75.68 (12.59)	75.68 (12.59)	Matching variable
30–49, years, n (%)	643 (3.52)	11,454 (16.43)	<0.001	596 (3.41)	596 (3.41)	Matching variable
50–64, years, n (%)	2985 (16.36)	18,533 (26.58)	<0.001	2873 (16.46)	2873 (16.46)	
65–79 years, n (%)	6404 (35.09)	18,552 (26.61)	<0.001	6114 (35.02)	6114 (35.02)	
≥80 years, n (%)	8218 (45.03)	21,187 (30.39)	<0.001	7874 (45.11)	7874 (45.11)	
CCI index. mean (SD)	0.82 (0.93)	0.5 (0.76)	0.000	0.82 (0.93)	0.63 (0.83)	<0.001
Obesity, n (%)	3838 (21.03)	7564 (10.85)	<0.001	3661 (20.97)	1773 (10.16)	<0.001
Myocardial infarction, n (%)	625 (3.42)	901 (1.29)	<0.001	602 (3.45)	321 (1.84)	<0.001
Congestive heart failure, n (%)	2702 (14.81)	5003 (7.18)	<0.001	2578 (14.77)	1771 (10.14)	<0.001
Peripheral vascular disease, n (%)	480 (2.63)	905 (1.3)	<0.001	462 (2.65)	295 (1.69)	<0.001
Cerebrovascular disease, n (%)	1014 (5.56)	2004 (2.87)	<0.001	974 (5.58)	729 (4.18)	<0.001
Dementia, n (%)	1884 (10.32)	5110 (7.33)	<0.001	1806 (10.35)	1821 (10.43)	0.792
COPD, n (%)	2139 (11.72)	8017 (11.5)	0.402	2051 (11.75)	2133 (12.22)	0.177
Rheumatoid disease, n (%)	561 (3.07)	2041 (2.93)	0.297	541 (3.1)	642 (3.68)	0.003
Mild Moderate/severe liver disease, n (%)	1042 (5.71)	2309 (3.31)	<0.001	1011 (5.79)	629 (3.6)	<0.001
Renal disease, n (%)	3673 (20.13)	5586 (8.01)	<0.001	3516 (20.14)	1861 (10.66)	<0.001
Cancer. or Metastatic cancer, n (%)	709 (3.88)	2581 (3.7)	0.245	675 (3.87)	712 (4.08)	0.311
Non-invasive mechanical ventilation, n (%)	932 (5.11)	2472 (3.55)	<0.001	904 (5.18)	682 (3.91)	<0.001
Invasive mechanical ventilation, n (%)	944 (5.17)	3213 (4.61)	0.001	897 (5.14)	760 (4.35)	0.001
Admission to ICU, n (%)	1364 (7.47)	4669 (6.7)	<0.001	1286 (7.37)	1074 (6.15)	<0.001
Day in ICU median (IQR)	10 (14)	10 (15)	0.230	10 (14)	10.5 (15)	0.294
LOHS. median (IQR)	9 (9)	7 (8)	<0.001	9 (9)	8 (8)	<0.001
IHM, n (%)	4229 (23.17)	10,339 (14.83)	<0.001	4079 (23.37)	3563 (20.41)	<0.001

* Matching was done 1 by 1 by age, place of residence, and month of admission. T2DM: type 2 diabetes mellitus; CCI: Charlson comorbidity index; COPD: chronic obstructive pulmonary disease; ICU Intensive care unit; IQR: Inter quartile range; LOHS: length of hospital stay; IHM: in-hospital mortality.

**Table 3:** Distribution of study covariates and hospital outcomes, before and after matching, for men hospitalized for COVID-19 in Spain in the year 2020 according to T2DM status.

	Before Matching			After Matching *		
	T2DM	No T2DM	*p*-Value	T2DM	No T2DM	*p*-Value
N (%)	27,370 (23.69)	88,142 (76.31)	<0.001	26,270 (50)	26,270 (50)	Matched 1:1
Age, mean (SD)	71.80 (12.28)	64.52 (15.79)	<0.001	71.67 (12.26)	71.67 (12.26)	Matching variable
30–49 years, n (%)	1305 (4.77)	17,447 (19.79)	<0.001	1261 (4.8)	1261 (4.8)	Matching variable
50–64 years, n (%)	6215 (22.71)	27,159 (30.81)	<0.001	6075 (23.13)	6075 (23.13)	
65–79 years, n (%)	11,700 (42.75)	25,192 (28.58)	<0.001	11,182 (42.57)	11,182 (42.57)	
≥80 years, n (%)	8150 (29.78)	18,344 (20.81)	<0.001	7752 (29.51)	7752 (29.51)	
CCI index, mean (SD)	0.92 (1.04)	0.54 (0.84)	<0.001	0.91 (1.04)	0.71 (0.92)	<0.001
Obesity, n (%)	4227 (15.44)	7583 (8.6)	<0.001	4087 (15.56)	2059 (7.84)	<0.001
Myocardial infarction, n (%)	2142 (7.83)	3155 (3.58)	<0.001	2050 (7.8)	1279 (4.87)	<0.001
Congestive heart failure, n (%)	3077 (11.24)	5027 (5.7)	<0.001	2936 (11.18)	2050 (7.8)	<0.001
Peripheral vascular disease, n (%)	1875 (6.85)	2824 (3.2)	<0.001	1811 (6.89)	1184 (4.51)	<0.001
Cerebrovascular disease, n (%)	1625 (5.94)	2745 (3.11)	<0.001	1528 (5.82)	1173 (4.47)	<0.001
Dementia, n (%)	1483 (5.42)	3178 (3.61)	<0.001	1399 (5.33)	1363 (5.19)	0.482
COPD, n (%)	4838 (17.68)	12,664 (14.37)	<0.001	4651 (17.7)	4780 (18.2)	0.143
Rheumatoid disease, n (%)	441 (1.61)	1087 (1.23)	<0.001	422 (1.61)	445 (1.69)	0.431
Mild Moderate/severe liver disease, n (%)	1943 (7.1)	4095 (4.65)	<0.001	1878 (7.15)	1252 (4.77)	<0.001
Renal disease, n (%)	5569 (20.35)	7246 (8.22)	<0.001	5315 (20.23)	2888 (10.99)	<0.001
Cancer, or Metastatic cancer, n (%)	1736 (6.34)	4555 (5.17)	<0.001	1676 (6.38)	1764 (6.71)	0.121
Non-invasive mechanical ventilation, n (%)	1867 (6.82)	4934 (5.6)	<0.001	1819 (6.92)	1748 (6.65)	0.218
Invasive mechanical ventilation, n (%)	2447 (8.94)	7712 (8.75)	0.330	2348 (8.94)	2337 (8.9)	0.866
Admission to ICU, n (%)	3405 (12.44)	10,589 (12.01)	0.059	3261 (12.41)	3177 (12.09)	0.264
Day in ICU median (IQR)	11 (16)	11 (18)	0.135	11 (16)	12 (19)	0.201
LOHS, median (IQR)	9 (9)	8 (9)	<0.001	9 (10)	9 (10)	0.904
IHM, n (%)	6657 (24.32)	14,826 (16.82)	<0.001	6375 (24.27)	5968 (22.72)	<0.001

* Matching was done 1 by 1 by age, place of residence and month of admission. T2DM: type 2 diabetes mellitus; CCI: Charlson comorbidity index; COPD: chronic obstructive pulmonary disease; ICU Intensive care unit; IQR: Inter quartile range; LOHS: length of hospital stay; IHM: in-hospital mortality.

**Table 4:** In-hospital mortality after matching for women and men with COVID-19 in Spain, 2020, according to T2DM status.

	Women *			Men *		
	T2DM	No T2DM	*p*-Value	T2DM	No T2DM	*p*-Value
N (%)	4079 (23.37)	3563 (20.41)	<0.001	6375 (24.27)	5968 (22.72)	<0.001
Age, mean (SD)	82.08 (9.22)	83.22 (8.49)	<0.001	78.3 (9.72)	79.31 (9)	<0.001
30–49, n (%)	16 (2.68)	8 (1.34)	0.106	49 (3.89)	16 (1.27)	<0.001
50–64, n (%)	198 (6.89)	114 (3.97)	<0.001	552 (9.09)	397 (6.53)	<0.001
65–79, n (%)	1099 (17.98)	847 (13.85)	<0.001	2556 (22.86)	2330 (20.84)	<0.001
≥80, n (%)	2766 (35.13)	2594 (32.94)	0.004	3218 (41.51)	3225 (41.6)	0.099
CCI index, mean (SD)	1.18 (1.01)	0.94 (0.92)	<0.001	1.31 (1.13)	1.1 (1.05)	<0.001
Obesity, n (%)	752 (20.54)	351 (19.8)	0.523	764 (18.69)	395 (19.18)	0.643
Myocardial infarction, n (%)	211 (35.05)	109 (33.96)	0.740	676 (32.98)	449 (35.11)	0.206
Congestive heart failure, n (%)	1000 (38.79)	688 (38.85)	0.969	1197 (40.77)	892 (43.51)	0.053
Peripheral vascular disease, n (%)	159 (34.42)	82 (27.8)	0.057	635 (35.06)	409 (34.54)	0.770
Cerebrovascular disease, n (%)	327 (33.57)	236 (32.37)	0.603	594 (38.87)	459 (39.13)	0.892
Dementia, n (%)	714 (39.53)	653 (35.86)	0.022	635 (45.39)	670 (49.16)	0.047
COPD, n (%)	453 (22.09)	406 (19.03)	0.015	1355 (29.13)	1323 (27.68)	0.117
Rheumatoid disease, n (%)	152 (28.1)	138 (21.5)	0.009	131 (31.04)	135 (30.34)	0.822
Mild Moderate/severe liver disease, n (%)	206 (20.38)	142 (22.58)	0.290	432 (23)	297 (23.72)	0.641
Renal disease, n (%)	1256 (35.72)	630 (33.85)	0.172	1969 (37.04)	1127 (39.02)	0.078
Cancer, or Metastatic cancer, n (%)	271 (40.15)	239 (33.57)	0.011	621 (37.05)	678 (38.44)	0.403
Non-invasive mechanical ventilation, n (%)	414 (45.8)	301 (44.13)	0.510	760 (41.78)	763 (43.65)	0.259
Invasive mechanical ventilation, n (%)	372 (41.47)	329 (43.29)	0.455	1108 (47.19)	1109 (47.45)	0.856
Admission to ICU, n (%)	468 (36.39)	396 (36.87)	0.810	1327 (40.69)	1285 (40.45)	0.841
Day in ICU median (IQR)	10 (16)	12.5 (16.5)	0.006	11 (18)	14 (19)	<0.001
LOHS, median (IQR)	6 (9)	7 (9)	0.002	8 (11)	8 (12)	<0.001

* Matching was done 1 by 1 by age, place of residence and month of admission. T2DM: type 2 diabetes mellitus; CCI: Charlson comorbidity index; COPD: chronic obstructive pulmonary disease; ICU Intensive care unit; IQR: Inter quartile range; LOHS: length of hospital stay; IHM: in-hospital mortality.

**Table 5:** Multivariable analysis of variables associated with in-hospital mortality in patients hospitalized with COVID-19 in Spain, 2020, according to sex and T2DM status.

	T2DM Women	T2DM Men	T2DM Both Sex	No T2DM Women	No T2DM Men	No T2DM Both Sex
	OR (CI 95%)	OR (CI 95%)	OR (CI 95%)	OR (CI 95%)	OR (CI 95%)	OR (CI 95%)
Age, 30–49 years	1	1	1	1	1	1
Age, 50–64 years	2.46 (1.14–5.33)	4.29 (2.54–7.26)	3.65 (2.37–5.63)	2.54 (1.46–4.39)	2.17 (1.57–2.99)	2.26 (1.71–2.99)
Age, 65–79 years	10.55 (4.99–22.31)	16.74 (9.98–28.08)	14.77 (9.65–22.59)	8.76 (5.14–14.95)	7.57 (5.53–10.36)	7.89 (6.02–10.34)
Age ≥80 years	41.66 (19.71–88.06)	60.53 (36.02–101.75)	54.91 (35.86–84.08)	25.60 (14.98–43.77)	23.06 (16.78–31.67)	23.65 (18.01–31.06)
Myocardial infarction	-	1.41 (1.23–1.61)	1.37 (1.22–1.54)	1.41 (1.17–1.70)	1.27 (1.14–1.42)	1.31 (1.19–1.44)
Congestive heart failure	1.64 (1.46–1.84)	1.62 (1.46–1.8)	1.63 (1.51–1.76)	1.55 (1.41–1.72)	1.52 (1.39–1.67)	1.53 (1.43–1.64)
Peripheral vascular disease	-	1.24 (1.08–1.43)	1.20 (1.06–1.36)	1.33 (1.07–1.64)	1.32 (1.18–1.48)	1.32 (1.20–1.46)
Cerebrovascular disease	1.40 (1.17–1.66)	1.60 (1.39–1.83)	1.52 (1.37–1.69)	1.43 (1.23–1.66)	1.72 (1.53–1.94)	1.60 (1.46–1.76)
Dementia	1.64 (1.46–1.84)	2.42 (2.14–2.74)	1.95 (1.80–2.12)	1.78 (1.59–1.99)	1.90 (1.68–2.14)	1.81 (1.67–1.97)
COPD	-	-	-	-	1.08 (1.00–1.17)	-
Rheumatoid disease,				1.34 (1.09–1.65)	-	1.20 (1.03–1.4)
Mild Moderate/severe liver disease	-	-	1.19 (1.04–1.35)	-	-	-
Renal disease	1.31 (1.17–1.47)	1.55 (1.42–1.71)	1.45 (1.35–1.56)	1.56 (1.43–1.71)	1.61 (1.49–1.73)	1.59 (1.50–1.68)
Cancer, or Metastatic cancer	2.54 (2.11–3.04)	2.33 (2.08–2.62)	2.40 (2.18–2.64)	2.79 (2.34–3.33)	1.90 (1.70–2.14)	2.13 (1.94–2.35)
Non-invasive mechanical ventilation	3.57 (2.96–4.31)	2.71 (2.41–3.06)	2.94 (2.65–3.25)	3.49 (2.97–4.09)	2.33 (2.07–2.62)	2.68 (2.44–2.95)
Invasive mechanical ventilation	2.75 (2.09–3.62)	3.21 (2.74–3.77)	3.03 (2.64–3.48)	2.78 (2.17–3.56)	2.92 (2.50–3.41)	2.88 (2.52–3.28)
Admission to ICU	1.61 (1.27–2.04)	1.69 (1.45–1.96)	1.61 (1.45–1.85)	1.49 (1.20–1.85)	1.75 (1.52–2.00)	1.67 (1.47–1.85)
Male sex	NA	NA	1.34 (1.27–1.42)	NA	NA	1.21 (1.15–1.28)

Only significant OR are shown in the table. T2DM: type 2 diabetes mellitus; COPD: chronic obstructive pulmonary disease; ICU: intensive care unit; NA: not applicable.

**Table 6:** Multivariable analysis of factors associated with in-hospital mortality in patients hospitalized with COVID-19 in Spain, 2020 according to sex.

	All Women	All Men	Both Sex
	OR (CI 95%)	OR (CI 95%)	OR (CI 95%)
Age, 30–49 years	1	1	1
Age, 50–64 years	2.53 (1.62–3.96)	2.68 (2.04–3.51)	2.63 (2.09–3.32)
Age, 65–79 years	9.56 (6.19–14.77)	9.81 (7.53–12.78)	9.81 (7.82–12.3)
Age ≥80 years	32.04 (20.72–49.54)	32.52 (24.91–42.46)	32.58 (25.96–40.90)
Myocardial infarction	1.34 (1.16–1.56)	1.33 (1.22–1.45)	1.33 (1.24–1.44)
Congestive heart failure	1.59 (1.47–1.71)	1.57 (1.46–1.68)	1.58 (1.5–1.66)
Peripheral vascular disease	1.20 (1.02–1.43)	1.29 (1.18–1.41)	1.27 (1.18–1.38)
Cerebrovascular disease	1.42 (1.27–1.59)	1.66 (1.52–1.82)	1.57 (1.46–1.68)
Dementia	1.71 (1.58–1.85)	2.14 (1.97–2.33)	1.88 (1.78–1.99)
COPD	-	1.06 (1.01–1.11)	-
Rheumatoid disease,	-	-	1.11 (1.00–1.24)
Mild Moderate/severe liver disease	-	1.13 (1.02–1.25)	1.12 (1.04–1.22)
Renal disease	1.46 (1.36–1.56)	1.58 (1.50–1.68)	1.53 (1.47–1.60)
Cancer, or Metastatic cancer	2.65 (2.33–3.00)	2.10 (1.94–2.28)	2.25 (2.11–2.41)
Non-invasive mechanical ventilation	3.54 (3.13–4.00)	2.51 (2.31–2.73)	2.80 (2.62–3.00)
Invasive mechanical ventilation	2.76 (2.30–3.32)	3.05 (2.73–3.41)	2.95 (2.68–3.25)
Admission to ICU	1.54 (1.12–1.82)	1.72 (1.54–1.89)	1.64 (1.52–1.799)
Male sex	NA	NA	1.27 (1.23–1.32)
T2DM	1.09 (1.03–1.16)	1.05 (1.01–1.10)	1.06 (1.03–1.10)

Only significant OR are shown in the table. T2DM: type 2 diabetes mellitus; COPD: chronic obstructive pulmonary disease; ICU: intensive care unit; NA: not applicable.

## Data Availability

According to the contract signed with the Spanish Ministry of Health and Social Services, which provided access to the databases from the Spanish National Hospital Database (RAE-CMBD, Registro de Actividad de Atención Especializada. Conjunto Mínimo Básico de Datos, Registry of Specialized Health Care Activities. Minimum Basic Data Set), we cannot share the databases with any other investigator, and we have to destroy the databases once the investigation has concluded. Consequently, we cannot upload the databases to any public repository. However, any investigator can apply for access to the databases by filling out the questionnaire available at http://www.msssi.gob.es/estadEstudios/estadisticas/estadisticas/estMinisterio/SolicitudCMBDdocs/Formulario_Peticion_Datos_CMBD.pdf (accessed on 25 April 2022). All other relevant data are included in the paper.

## Supplementary Materials

The following supporting information can be downloaded at: https://www.mdpi.com/article/10.3390/jcm11092654/s1, Table S1: ICD-10 codes for diagnosis and therapeutic procedures used in this investigation.
