# Supplementary material for: Impact of Type 2 Diabetes Mellitus on the Incidence and Outcomes of COVID-19 Needing Hospital Admission According to Sex: Retrospective Cohort Study Using Hospital Discharge Data in Spain, Year 2020
Source: J Clin Med. 2022 May 9;11(9):2654. doi: 10.3390/jcm11092654 (PMC9104185; doi:10.3390/jcm11092654)
Supplement: Supplementary file 1 [file jcm-11-02654-s001.zip › jcm-1675206-supplementary.pdf]

Supplemental Table S1. ICD-10 codes for diagnosis and therapeutic procedures used in this investigation.

Description		ICD-10 codes
Codes when a coronavirus has been identified	Coronavirus infection, unspecified	B34.2
	Other coronavirus as the cause of diseases classified elsewhere	B97.29
	COVID 19	U07.1
Beside any of the three codes for a coronavirus, one or more of these conditions must be codified	Viral pneumonia, unspecified	J12.9
	Other viral pneumonia	J12.89
	Acute bronchitis due to other specified organisms	J20.8
	Acute bronchitis, unspecified	J20.9
	Unspecified acute lower respiratory infection	J22
	Other specified respiratory disorders	J98.8
	Acute respiratory distress syndrome	J80
	Severe sepsis without septic shock	R65.20
	Severe sepsis with septic shock	R65.21
Type 2 diabetes *		E11.xxx
Type 1 diabetes (exclusion)		E10.xxx
Obesity		E66.09, E66.1, E66.2, E66.01E66.3, E66.8 E66.9
Non-invasive mechanical ventilation		5A09357, 5A09457, 5A09557
Invasive mechanical ventilation		5A1945Z, 5A1955Z, 5A1935Z

*Each discharge diagnosis has a "Present on Admission (POA)" indicator assigned according to the ICD-10-CM Official Guidelines for Coding and Reporting (**https://icdlist.com/icd-10/guidelines/**). The reporting options and definitions for POA are "Y" (present at admission); "N" (not present at admission); "U" (lack documentation to determine presence at admission); "W" (provider is unable to clinically determine if the condition was present); and unreported/not used. Only T2DM patients with "Y" were included in the study population.

The ICD 10 codes for conditions included in the Charlson Comorbidity Index can be found in references

Sundararajan V, Henderson T, Perry C, Muggivan A, Quan H, Ghali WA. New ICD-10 version of the Charlson comorbidity index predicted in-hospital mortality. J. Clin. Epidemiol 2004;57:1288–94. doi: 10.1016/j.jclinepi.2004.03.012.

Quan H, Sundararajan V, Halfon P, Fong A, Burnand B, Luthi JC, et al. Coding algorithms for defining comorbidities in ICD-9-CM and ICD-10 administrative data. Med Care. 2005;43:1130-9. doi: 10.1097/01.mlr.0000182534.19832.83.
